# Predicting the 10-year risk of cardiovascular diseases and its relation to healthy diet indicator in Iranian military personnel

**DOI:** 10.1186/s12872-021-02231-y

**Published:** 2021-09-05

**Authors:** Karim Parastouei, Mojtaba Sepandi, Eslam Eskandari

**Affiliations:** Health Research Center, Life Style Institute, Baqiyatallah University of Medical Sciences, Tehran, Iran

**Keywords:** Cardiovascular disease, Healthy diet indicator, Framingham risk score

## Abstract

**Background:**

Epidemiological studies indicate increased prevalence of cardiovascular disease (CVD) among military personnel. Accordingly, identification of at-risk individuals and lifestyle modification such as improving diet quality can potentially inhibits the increasing trend of CVD mortality. The aim of this study was predicting the 10-year risk of CVD and its association with healthy diet indicator (HDI) among military personnel.

**Methods:**

In this cross-sectional study, 400 male military personnel within the age range of 30–75 years were included. HDI score was calculated based on food frequency questionnaire, and the 10-year risk of CVD was evaluated using Framingham risk score (FRS). The FRS items include age, gender, total cholesterol, high density lipoprotein cholesterol (HDL-C), systolic blood pressure, status of diabetes and smoking. Partial correlation test was employed to investigate the relationship between Framingham risk score and HDI score.

**Results:**

The mean age and body mass index (BMI) of participants were 38.67 ± 5.3 year and 25.28 ± 3.22 kg/m^2^, respectively. Prediction of FRS was as follows: 96.5% were low risk, 2% were moderate risk, and 1.5% were high risk. The mean HDI score of participants in this study was 5.98 ± 1.36. While HDI score did not show a significant correlation with FRS (r: − 0.009, *p*:0.860), increased dietary sodium intake had a significant positive correlation with FRS (r: 0.114, *p*:0.026).

**Conclusion:**

The most of participants (96.5%) had in low risk of CVD development in the next 10 years. Meanwhile, the FRS showed no significant relationship with HDI score. Further researches are required to confirm the results of the present study.

## Introduction

Cardiovascular disease (CVD) is an important cause of mortality in both developed and developing countries [1, 2]. CVD led to 17 million deaths in 2013 [3]. It has been estimated that mortality from CVD would reach 23.6 million worldwide by the end of 2030 [4]. Also, previous evidence suggests ascending trend of CVD prevalence in Iran; epidemiological studies indicate that total mortality caused by CVD would reach 44.8% by 2030 in Iran [5]. The most important risk factors for CVD include unhealthy diet, hypertension, tobacco smoking, dyslipidemia, obesity, glucose disorders, and stress [6, 7]. Occupational stress can significantly affect the prevalence of CVD [8]. For example, military-related stressors are correlated with acute cardiac disorders [9]. In this regard, there are an increased prevalence of CVD as well as its risk factors in military personnel [10–12]. Previous studies have found that the prevalence of hypertension and hypertriglyceridemia among the military personnel is 8.8 and 30.5% in Iran, respectively [9].

Previous findings also suggest an inverse relationship between diet quality and CVD prevalence [13]. Consumption of some foods such as fish, fruits and vegetables can reduce the risk of CVD development [14]. Based on the theory of diet-diseases reaction, different dietary compounds have various effects on progression or prevention of diseases. Accordingly, investigating of the general dietary intake quality is prefered over examining individual intake of every single food item [15]. Based on the world health organization (WHO) guidelines, indicators for dietary quality measurement are considered as a tool for investigation of diet quality in different societies. Healthy dietary index (HDI) was also introduced in the 1990s as one of these dietary quality indicators to prevent chronic diseases [16]. Previous reports suggest an inverse association between HDI and all-cause mortality in adults. In this regard, findings of Berentzen et al*.* study showed groups with higher HDI score had18% reduction in risk of CVD mortality [17]. On the other hand, limited studies have been performed to predict the CVD risk and its association with HDI score among military personnel. Among different risk scores were used to estimate the risk of CVD Framingham risk score (FRS) is a common and simple tool. This score predicts the risk of CVD development in next 10-year based on the data related to age, gender, total cholesterol, status of diabetes, systolic blood pressure, high density lipoprotein cholesterol (HDL-C), and tobacco smoking [18].

Considering the increasing prevalence of CVD in Iran and the lack of studies conducted on 10-year risk assessment of CVD as well as its association with dietary quality using HDI-2020 score among the military personnel, the aim of the present study was to predict the 10-year risk of CVD development using FRS and its relationship with HDI score.

## Materials and methods

### Study design and participants

The present cross-sectional study was performed on male military personnel in September 2020. Considering a frequency (prevalence) of 0.50 and type one error of 0.05 the sample size of 400 was calculated. Random cluster sampling was done among four clusters (groups) of the military personnel living in Tehran province. The inclusion criteria were as follows: willingness to participate in study, age range 30–75 years, no CVD disease, and no food allergy. The exclusion criteria were following a certain diet and lack of cooperation for required information in the research.

### Basic data on biochemical analysis

The basic information included anthropometric measurements (body mass index (BMI), height and weight), age, physical activity, smoking habit, diabetes, systolic blood pressure, total cholesterol, and HDL-C was gathered for all participants. The weight was measured with less clothing and bare feet using a standard digital balance with accuracy of 100 g. The height was measured using a band tape with no shoes and accuracy of 0.5 cm. BMI, was calculated based on the formula (weight(kg)/height(m)^2^). Data about physical activity level (more than 30 min/day or less than 30 min/day), cigarette smoking (yes/no), and diabetes mellitus (yes/no) were collected by face-to-face interview. Blood pressure measurement was performed using a standard sphygmomanometer (Microlife BP AGI-20/Switzerland) in sitting state from the right arm of participants by an experienced physician. Blood samples were taken after 12 h fasting to determine the laboratory data. Samples were centrifuged at 2500 rpm for 15 min. Fasting plasma glucose (FPG) was measured using enzymatic-colorimetric glucose oxidase method. The total cholesterol was measured using enzymatic method based on cholesterol esterase and cholesterol oxidase. Also, HDL-C was measured specifically using the antibodies functioning against human lipoproteins, with the same enzymatic method used for the total cholesterol.

### Dietary assessment and HDI score

The 168-item semi-quantitative food frequency questionnaire was employed to investigate the usual dietary intake of the participants. The reliability and validity of this questionnaire had already been assessed in previous studies [19]. Through the face-to-face interview by experienced experts, each of the participants was asked to report the frequency of consuming each food item per day, week, month, or year based on the standard portion sizes. The obtained values of were converted to gram per day using household measures. Finally, the daily consumption values for each food item were determined. We used the United States Department of Agriculture (USDA) pyramid for determination of consumed servings (gr) of each food group. Daily energy expenditure and nutrient intake was determined by N4 software. We used the updated version (HDI-2020) of dietary recommendations for prevention of chronic disease published by the World Health Organization (WHO) to cover the last recommendations. Investigation of the dietary quality by HDI-2020 has been assessed in the previous studies [20]. HDI listhas eleven items with the score range of 0–11. Zero represents minimum adherence of diet to HDI, while 11 indicates the maximum adherence to HDI. Individual components of HDI have a specific range and can vary between 0 and 1. Intake of fruits and vegetables, beans and legumes, nuts and seeds, dietary fiber, whole grains, sodium, total fat, free sugars, saturated fatty acids (SFA), processed meat, and unprocessed red meat were scored by the index of interest. Generally, the recommendations suggest increasing consumption of five main items and limiting the consumption of the six other items in daily diet. The details of HDI-2020 scoring components are shown in Table 1.

**Table 1:** Healthy diet indicator elements (HDI-2020)

Dietary component	Criteria for scoring	Scoring*
Whole grains	> 0 g/day	0/1
Beans and other legumes	> 0 g/day	0/1
Fruits, vegetables	≥ 400 g/day	0/1
Nuts and seeds	> 0 g/day	0/1
Dietary fiber	> 25 g/day	0/1
Total fat	< 30% total energy/day	0/1
Saturated fat	< 10% total energy/day	0/1
Free sugars	< 10% total energy/day	0/1
Dietary sodium	< 2 g /day	0/1
Unprocessed red meat	≤ 71 g/day	0/1
Processed meat	0 g/day	0/1

*Total index score; minimum: 0, maximum: 11. If perent or quantities are not in the ranges, score = 0

### CVD risk assessment

The 10-year risk prediction of CVD was performed by FRS. FRS were determined based on the six CVD risk factors in men including age, HDL-C, systolic hypertension (treated/untreated), total cholesterol, smoking habits, and status of diabetes. FRS items have a specific score range (Table 2). For example, diabetic patients received score 3, while the subjects without diabetes were scored zero. Also, score 4 was assigned to smokers, while 0 was dedicated to those with no smoking habit. After calculating the score for each of the mentioned factors in Table 2, the sum of these scores generated the total FRS. The range of the total FRS can be ≥  − 3 up to ≤ 21. The 10-year risk percentage of CVD, calculated according to the total FRS (-3 or less points: < 1%, − 2 points: 1.1%, − 1 point: 1.4%, 0 point: 1.6%, 1 point: 1.9%, 2 points: 2.3%, 3 points: 2.8%, 4 points: 3.3%, 5 points: 3.9%, 6 points: 4.7%, 7 points: 5.6%, 8 points: 6.7%, 9 points: 7.9%, 10 points: 9.4%, 11 points: 11.2%, 12 points: 13.3%, 13 points: 15.6%, 14 points: 18.4%, 15 points: 21.6%, 16 points: 25.3%, 17 points: 29.4%, 18 points or more: > 30). The 10-year CVD risk grade can be as follows: low; FRS < 10%, moderate; FRS = 10–19%, and high; FRS ≥ 20% [21].

**Table 2:** The cutoffs of CVD risk factors for calculating Framingham risk score in men

Age (year)	HDL-C	Total cholesterol	SBP not treated	SBP treated	Smoker	Having diabetes	Risk point for each factor
	+ 60		˂ s120				− 2
	50–59						− 1
30–34	45–49	˂ 160	120–129	˂ 120	No	No	0
	35–44	160–199	130–139				1
35–39	˂ 35	200–239	140–159	120–129			2
		240–279	+ 160	130–139		Yes	3
		+ 280		140–159	Yes		4
40–44				+ 160			5
45–49							6
							7
50–54							8
							9
55–59							10
60–64							11
65–69							12
							13
70–74							14
+ 75							15

*SBP* Systolic blood pressure; *HDL-c* high-density lipoprotein-cholesterol

### Statistical analysis

Comparison of categorical variables across the Framingham risk subgroups was performed by chi-square test. This comparison was done by Kruskal–Wallis and one-way Analysis of variance (ANOVA) test for continuous variables. Normality and variance of data (homogeneity of variances) were examined by Shapiro Wilk test as well as Leven's statistics respectively. The relationship between HDI score and its items with FRS was assessed by partial correlation test. Age, daily energy intake, smoking habit, physical activity, BMI, waist circumference (WC), hip circumference (HC), waist hip ratio (WHR), and history of diabetes were also controlled in order to examine the mentioned relationship. These variables were reported based on mean ± SD, percentage, median, and interquartile range (IQR). All statistical analyses were done in SPSS 22 software, with the significance level of less than 0.05.

## Results

The mean age and BMI of the participants were 38.76 ± 5.3 year and 25.28 ± 3.22 kg/m^2^, respectively. Also, 52% of the participants had BMI > 25 kg/m^2^ and the rest had BMI ≤ 25 kg/m^2^. The mean WC, HC, and WHR were 46.34 ± 4.19 cm, 49.54 ± 4.21 cm, and 0.93 ± 0.05 cm respectively. Furthermore, 4.2% of the participants were smoker, while 95.8% had no smoking habit. The level of physical activity was suitable in most participants; About 79.7% of them had moderate and high physical activity level, while only 20.3% reported low physical activity (less than 30 min /day). Generally, the mean daily calorie intake among all participants was 2285.11 ± 1028.68 kcal/day. No one reported alcohol consumption among the all participants. Based on Framingham model,, 96.5% of subjects had low risk, 2% had moderate risk, and 1.5% had high-risk for 10-year CVD risk development. Also, the mean HDI score of the participants was 5.98 ± 1.36.

Data about anthropometric indicators, lifestyle, taking medicine, diseases history, biochemical analysis, dietary intake, systolic blood pressure, and mean HDI score were reported in Table 3 among the FRS categories. Table 3 indicates that more senior individuals have a higher 10-year CVD risk level (*p* < 0.001). Also, the participants without diabetes and non-smokers fell into the low-risk population group (*p* < 0.001). In moderate risk group, systolic blood pressure and FPG were significantly higher compared with the two other groups (*p*:0.033, 0.010). On the other hand, 95% of the participants had untreated high systolic blood pressure (hypertension). Meanwhile, HDI score was not significantly different across the low, moderate, and high-risk groups (*p*-value = 0.940). Also, those with low FRS had a higher rate of processed meat consumption (*p* = 0.002). On the other hand, calorie intake as well as other nutrients or food groups including whole grains, beans and other legumes, fruits and vegetables, nuts and seeds, dietary fiber, total fat, SFA, free sugars, dietary sodium, and unprocessed meat did not differ significantly across the three study groups (low, moderate, and high-risk) (*p* > 0.05) (Table 3).

**Table 3:** Characteristics of the study population according to Framingham risk level

Variables^†^	Total	FRS			*p*-value
		Low (< 10%)	Intermediate (10–19%)	High (> 20%)	
No. of participants, n (%)	400 (100)	386 (96.5)	8 (2)	6 (1.5)	
Age (year), n (%)					
30–50 years	389 (97.3)	382 (98.2)	6 (1.5)	1 (0.3)	< 0.001^b^
50–75 years	11 (2.8)	4 (36.4)	2 (18.2)	5 (45.5)	
Body mass index (kg/m^2^)	25.28 ± 3.22	25.19 ± 3.13	28.22 ± 6.14	26.95 ± 1.98	0.158^a^
Waist circumference (cm)	46.34 ± 4.19	46.33 ± 4.17	45.75 ± 5.31	48.16 ± 4.4	0.484^a^
Hip circumference (cm)	49.54 ± 4.21	49.52 ± 4.20	48.37 ± 5.15	52.16 ± 3.12	0.109^a^
Waist–hip ratio	0.93 ± 0.05	0.93 ± 0.05	0.94 ± 0.02	0.92 ± 0.03	0.563^a^
Physical activity level, n (%)					
Low	81 (20.3)	78 (96.3)	2 (2.5)	1 (1.2)	0.923^b^
Moderate/high	319 (79.7)	308 (96.6)	6 (1.9)	5 (1.6)	
Drug uses (%)					
No	301 (75.3)	289 (96)	6 (2)	6 (2)	0.367^b^
Yes	99 (24.8)	97 (98)	2 (2)	0 (0)	
Diseases history (except for diabetes) (%)					
No	308 (77)	296 (96.1)	7 (2.3)	5 (1.6)	0.720^b^
Yes	92 (23)	90 (97.8)	1 (1.1)	1 (1.1)	
Smoking status, n (%)					
No	383 (95.8)	377 (98.4)	5 (1.3)	1 (0.3)	< 0.001^b^
Yes	17 (4.2)	9 (52.9)	3 (17.6)	5 (29.4)	
Having diabetes, n (%)					
No	384 (96)	377 (98.2)	3 (0.8)	4 (1)	< 0.001^b^
Yes	16 (4)	9 (56.3)	5 (31.3)	2 (12.5)	
SBP (mmHg)	121.25 ± 9.38	121.02 ± 9.35	128.75 ± 6.4	125.83 ± 10.2	0.033^c^
FPG (mg/dl)	117.88 ± 13.52	117.62 ± 13.10	132.04 ± 19.84	115.48 ± 22.13	0.010^c^
HDL (mg/dl)	43 ± 7.01	43.09 ± 6.98	41.76 ± 9.01	38.84 ± 4.87	0.299^c^
TC (mg/dl)	150.83 ± 21.56	150.48 ± 21.18	163.16 ± 38.36	156.70 ± 13.81	0.206^c^
HDI score	5.98 ± 1.36	5.99 ± 1.37	5.87 ± 1.12	5.83 ± 1.47	0.940^c^
Dietary intake, median (IQR)					
Energy intake (kcal/day)	2027.4 (1303.35)	2024.04 (1324.43)	2130.04 (1722.31)	2400.72 (924.50)	0.641^a^
Whole grains (g/day)	208.37 (146.86)	206.64 (142.23)	325.55 (196.43)	299.01 (166.75)	0.158^a^
Beans and other legumes (g/day)	28.12 (23.37)	27.79 (23.06)	27.90 (30.18)	31.67 (33.76)	0.947^c^
Fruits, vegetables (g/day)	842.85 (845.64)	832.49 (857.11)	922.21 (797.44)	1094.94 (730.66)	0.957^c^
Nuts and seeds (g/day)	19.23 (24.39)	19.33 (27.58)	17.69 (12.5)	19.24 (4.93)	0.791^c^
Dietary fiber (g/day)	28.7 (21.03)	28.7 (21.30)	29.08 (23.26)	38.96 (21.91)	0.967^c^
Total fat (% total energy)	32.45 (7.36)	32.3 (7.64)	33.73 (8.84)	32.96 (6.99)	0.936^a^
Saturated fat (% total energy)	10.3 (3.73)	10.36 (3.69)	10.13 (4.49)	10.35 (6.20)	0.982^a^
Free sugars (% total energy)	43.49 (15.97)	43.52 (16.24)	43.78 (21.18)	44.5 (19.92)	0.790^a^
Dietary sodium (g/day)	3.44 (2.84)	3.42 (2.76)	6.48 (6.27)	8.06 (3.94)	0.927^a^
Unprocessed red meat (g/day)	73.19 (54.64)	73.19 (55.14)	85.92 (67.93)	81.52 (34.34)	0.448^a^
Processed meat (g/day)	13.12 (15)	13.75 (15.54)	8.04 (8.44)	9.01 (12.54)	0.002 ^a^

*FRS* Framingham risk score, *FPG* Fasting plasma glucose, *SBP* Systolic blood pressure, *TC* Total cholesterol; *HDL-c*; high-density lipoprotein-cholesterol, *HDI* Healthy diet indicator

^a^Kruskall–Wallis test

^b^Chi-square test

^c^One-way ANOVA test

^†^All values are expressed as mean ± standard deviation (SD), median (interquartile range) and number (percent)

*p-value < 0.05 considered as significant level

Partial correlation of FRS with HDI items among participants were outlined in Table 4. Although HDI score showed an inverse correlation with FRS, this relationship was not significant (r: − 0.009, *p*:0.860). On the other hand, increased dietary sodium intake lead to a significant increment in FRS level (r:0.114, *p*:026), and consequently, more CVD risk in the next 10 years. Other HDI elements showed no significant correlation with FRS (*p* > 0.05). These findings were adjusted for confounding variables including age, energy intake, smoking habit, physical activity, BMI, WC, HC, WHR, history of diabetes, taking medicine and diseases history (other than diabetes) (Table 4).

**Table 4:** Partial correlation of Framingham risk score (FRS) with HDI items among participants

Variables^©^	FRS		
	Median (IQR)	r	p*
Whole grains (g/day)	208.37 (146.86)	− 0.056	0.272
Beans and other legumes (g/day)	28.12 (23.37)	0.045	0.376
Fruits, vegetables (g/day)	842.85 (845.64)	− 0.016	0.754
Nuts and seeds (g/day)	19.23 (24.39)	− 0.070	0.171
Dietary fiber (g/day)	28.7 (21.03)	− 0.023	0.652
Total fat (% total energy)	32.45 (7.36)	− 0.026	0.614
Saturated fat (% total energy)	10.3 (3.73)	− 0.020	0.702
Free sugars (% total energy)	43.49 (15.97)	0.019	0.715
Dietary sodium (g/day)	3.44 (2.84)	0.114	0.026
Unprocessed red meat (g/day)	73.19 (54.64)	− 0.023	0.652
Processed meat (g/day)	13.12 (15)	− 0.009	0.865
Total HDI score	6 (2)	− 0.009	0.860

©Adjusted for total energy intake, body mass index, age, physical activity, waist circumference, hip circumference, waist–hip ratio, diseases history (other than diabetes), drug uses, history of diabetes disease and smoking status

*p value < 0.05 considered as significant level

## Discussion

The present study found that 96.5% of the military personnel participated in the study had FRS less than 10%. On the other hand, 2% of these people showed moderate risk (10–19%) and 1.5% had a risk above 20%. Furthermore, after adjustment for confounding variables, HDI score had no meaningful relationship with FRS, but dietary sodium intake indicated a considerable positive relationship with FRS.

Population-based epidemiological studies suggest that prevalence of CVD as well as its risk factors are growing among the military personnel [11]. Meanwhile, past evidence suggest that controlling CVD risk factors can considerably reduce development and mortality of CVD across the military population [12]. In this regard, various prospective studies have been conducted worldwide to predict the risk of developing CVD across the military population. Findings of the present study showed that a wide range (96.5%) of the military personnel participated in this study had a low risk of CVD for the next 10-year. Accordingly, the study by Al-Dahi et al*.* performed on 10,500 military personnel in Saudi Arabia also showed that only 9.1% of participants had a 10-year risk of CVD above 10%, while 90.9% of the participants showed a risk lower than 10%. The mean FRS across the military personnel in this study was 4.5 [22].

On the other hand, prevalence of CVD risk factors and 10-year risk of CVD among the Belgian military men was examined by Mullie et al*.* (2010). Prevalence of obesity among the official officers, unofficial officers, and soldiers was 5.6%, 15%, and 19.5%, respectively. Only 8.5% of the officers had a 10-year risk of CVD above 5%. Also, high-risk participants were younger than 40 year, and 12.5% of official officers and 19.7% of unofficial officers had smoking habits [23]. However, the results of the study by Grósz et al*.* were inconsistent with the mentioned studies. Grósz et al*.* determined the 5-year risk of CVD among 250 male military pilots. They found that half of the participants had a five-year risk of CVD above 2.5%; this number did not exceed 15–20% even in the high-risk age group. In addition, the prevalence of CVD risk factors among the military pilots was as follows: insufficient physical activity: 23.9%, smoking: 31.7%, high blood pressure: 14.7%, and obesity: 40.8% [24]. The discrepancies across these studies can be examined from different aspects. Previous evidence showed that smoking can considerably increase risk of CVD. Prevalence of smoking in the present study was only 4.2%. Also, the 10-year risk of CVD was lower meaningfully. However, in the study by Grósz et al. the prevalence of smoking was around 31.7%, and hence the five-year risk of CVD was considerably higher. Furthermore, the mean BMI in our study was about 25 kg/m^2^, while 48.8% of the military pilots in the Grósz et al*.* study had BMI > 25 kg/m2, and 40.8% of the participants considered as obese. In addition, compared to the previous studies with discrepant results, the military personnel participated in the current study had more desirable status regarding the blood pressure and total cholesterol. Thus they showed lower FRS [23, 24]. Based on the mentioned justifications, it seems that the results obtained in the present study are reasonably acceptable.

The results showed that the HDI-2020 had no meaningful relationship with FRS among the military personnel, but increased dietary sodium intake -as one of the HDI elements- led to a considerable rise in FRS. Although there are so limited studies on dietary quality measurement using HDI among the military personnel, some researchers have conducted notable studies on examining the relationship between HDI score and CVD risk or mortality among other population groups. In accordance to the present study, Mertens et al*.* studied 1867 healthy men. They found no meaningful relationship between HDI and incidence of CVD. However, increase in HDI score had an inverse considerable relationship with C-reactive protein (CRP). CRP is one of the major risk factors in CVD. On the other hand, intake of calorie, protein, carbohydrate, sodium, fiber, and fat had no meaningful difference across the HDI score subgroups [25]. A cohort study by Struijk et al*.* on 33,671 healthy men and women in Netherlands showed no considerable relationship between HDI and CVD risk [26]. Furthermore, the results of Atkins et al*.* on 3325 British men (aged 60–79 year) suggested that the mortality risk caused by CVD had no meaningful relationship with HDI score. Also, increased consumption of saturated fatty acids (SFA), poly unsaturated fatty acids (PUFA), protein, carbohydrate, sugar, fiber, fruits and vegetables had no considerable effect on the CVD incidence. However, increased dietary cholesterol intake, as one of the HDI items, increase the CVD mortality [27]. On the other hand, Stefler et al*.* showed that higher HDI score was associated with a considerable reduction in CVD mortality among 1855 elderly people in the Eastern Europe [14]. Also, Knoops et al*.* reported that higher HDI score had an inverse relationship with all-cause and CVD mortality risk [28]. We can discuss about these discrepancies by means of different assumptions. One of these assumptions is related to the type of items examined by HDI-2020 in the present study. Some past researches with inconsistent results had dealt with the original HDI version, while our study conducted on the updated version of this index. One of the differences between these two versions is related to dietary sodium intake, which had not been mentioned in the original HDI version, but the present study had considered the daily sodium intake as one of the items in HDI score calculation. Meanwhile, some previous researches showed that increase in consumption of some food items/groups such as fruits, vegetables, fiber, and whole grains can reduce the risk of CVD, but the results of the present study did not confirm this claim. One of the mentioned assumptions can be due to the insufficient consumption of these foods by Iranian adults. Although there are a comprehensive dietary guideline for Iranian population, it seems some changes have occurred in the Iranian dietary habits [4].

Another justification is related to the gender of participants. Based on the previous studies, sex have no effect on the association between HDI score and FRS. In this regard, the results of Huffman et al. confirmed this rationale. The study of Huffman et al*.* indicated that gender have no effect on the association between diet quality and CVD risk in type 2 diabetes mellitus patients. However, the study by Stefler et al*.* did not confirm this claim. The results of Stefler et al*.* indicated that women have a higher HDI score compared to men [16]. Considering the gender of the participants in the current study, it seems that HDI score may be not adequately high across the male military personnel. Thus, the low range of HDI score in the present study has resulted from the minor influence of this index on the 10-year risk of CVD. Due to the limited number of studies assessing the relationship between dietary quality indices and 10-year CVD risk among military personnel, the effect of gender on this relationship cannot definitively rule out. It needs further studies to investigate this effect.

The main limitation of the present study was its cross-sectional design, which can complicate extraction of causal relations between HDI and 10-year risk of CVD. Another limitation was gender of participants. Since the female military personnel claims a very minor percentage of the Iranian military community, thus the participants were chosen from male personnel. Also, considering the military participants in the study and the study location, we had some limitations for collecting more detailed data. The strong points of this study were as follows: 1. We adjusted the results for the most important confounders. So, this can increase the validity of the results. 2) To the best of our knowledge, there was no previous studies have assessed the relationship between FRS and HDI score among the military personnel in Iran, and this study can be regarded as the first study in this field in Iran.

## Conclusion

The results of this cross-sectional study showed that a wide range of the military personnel participated in this study had a low risk of CVD development in the next 10 years; About 96.5% had FRS of less than 10%, and only 1.5% of them had a risk higher than 20%. Meanwhile, after dietary assessment of the participants, the results showed that the dietary quality measured by HDI had no meaningful relationship with FRS. However, increased sodium intake considerably increased the risk of CVD development in the next 10 years. Also, higher sodium intake was direct meaningful correlation with FRS.

## Data Availability

The datasets generated and/or analyzed during the current study are not publicly available due to the importance of study population (military personnel), but are available from the corresponding author on reasonable request.
